# Enhanced and proficient chitosan membranes embedded with polyaniline-TiO_2_ core-shell nanocomposites for fuel-cell hydrogen storage

**DOI:** 10.55730/1300-0527.3730

**Published:** 2024-12-25

**Authors:** Mallikarjunagouda B. PATIL, Shridhar N. MATHAD, Arun Y. PATIL, Abdulaziz Abdulah Al-KHERAIF, Sachin NAIK, Sajith VELLAPALLY

**Affiliations:** 1 Membrane Separation Division, Bharat Ratna Prof. C.N.R. Rao Research Centre, Basaveshwar Science College, Bagalkot, Karnataka, India; 2 Department of Engineering Physics, K.L.E. Institute of Technology, Hubballi, India; 3 Department of Mechanical Engineering, Manipal Institute of Technology-Bengaluru, Manipal Academy of Higher Education, Manipal, Karnataka, India; 4 Department of Dental Health, College of Applied Medical Sciences, King Saud University, Riyadh, Saudi Arabia

**Keywords:** Metal oxide nanoparticles, polyaniline, core-shell, direct current conductivity, hydrogen storage

## Abstract

This study investigates the preparation and properties of aniline polymerized in situ onto a nanosized TiO_2_ surface to form core-shell nanoparticles at ambient temperatures. The in situ polymerization of aniline to polyaniline (PANI), in conjunction with the utilization of an anionic surfactant, was employed in this investigation. The prepared PANI-TiO_2_ core-shell nanoparticles were integrated with chitosan at a gravimetric ratio and cast as core-shell nanocomposite membranes. The nanocomposites were subjected to structural analysis using Fourier transform infrared spectroscopy and X-ray diffraction patterns. The surface morphologies of the PANI and its nanocomposites were analyzed using scanning electron microscopy. Direct current conductivity studies revealed three discrete tiers of conductivity intrinsic to a semiconductor material. The nanocomposite, comprising a chitosan membrane embedded with 4 wt.% PANI-TiO_2_, demonstrated peak direct current conductivity of 5.7 S/cm. The properties of the core-shell nanocomposite membranes could be elucidated using cyclic voltammetry, a technique that allowed for the observation of redox peaks occurring at 0.94 V and 0.25 V. The presence of both peaks was due to the redox transition of the prepared nanocomposite membranes from a semiconducting to a conductive state. At room temperature, the hydrogen absorption capacity was approximately 4.5 wt.%, but when the temperature was raised to 65 °C, it doubled to about 7.5 wt.%. In comparison to other nanocomposites, the 4 wt.% PANI-TiO_2_ core-shell embedded chitosan membrane exhibited significantly higher absorption capacity of 10.5 wt.%.

## 1. Introduction

Hydrogen is often considered a clean fuel due to several key attributes that make it an attractive option for a future based on sustainable energy [1]. These attributes include zero emissions, high energy content, renewable production, energy storage capabilities, versatile applications, decentralized production, and reduced dependence on fossil fuels [2,3]. However, it is important to note that hydrogen is not without challenges, including production emissions, storage and transportation challenges, safety concerns, and issues of economic viability. Overall, hydrogen has the potential to be a clean fuel when produced using renewable methods and it can play a vital role in reducing greenhouse gas emissions and transitioning to more sustainable energy systems in the future. However, addressing challenges related to cost, safety, and infrastructure will be essential for realizing the full potential of hydrogen as a clean energy carrier [4,5].

In the pursuit of a more sustainable and environmentally responsible energy landscape, the necessity of hydrogen storage cannot be overstated [6,7]. Hydrogen, often hailed as the “fuel of the future,” possesses immense potential as a clean energy carrier. It is a versatile solution for addressing a myriad of global challenges, including the needs of reducing greenhouse gas emissions, achieving energy security, and transitioning away from fossil fuels. However, the gaseous nature of hydrogen under standard conditions presents a significant hurdle that must be overcome to fully unlock its potential [8–10]. Hydrogen is a clean and efficient energy source that can be produced from a variety of sources, including water and renewable energy sources such as wind and solar power. When used in fuel cells or combustion engines, hydrogen generates electricity with zero emissions, producing only pure water as a byproduct. This unique attribute makes it critical in the fight against climate change and air pollution, offering a way to mitigate environmental damage and improve air quality [11–14]. To harness hydrogen’s potential, efficient and safe storage methods are imperative. The diverse applications of hydrogen in sectors such as transportation, power generation, and industrial processes require a range of storage solutions to meet specific needs. Compressed gas, liquefied hydrogen, metal hydrides, chemical hydrogen storage, and solid-state materials are just a few of the avenues under exploration [15].

One of the driving forces behind the development of effective hydrogen storage is the need for energy storage and balance in a world increasingly reliant on intermittent renewable energy sources like wind and solar power. Hydrogen serves as a means of storing excess energy during periods of high production and releasing it when demands surge. This capability is integral to achieving energy grid stability and reliability. Moreover, hydrogen can contribute to the reduction of greenhouse gas emissions in sectors that are challenging to decarbonize, such as heavy industry and long-haul transportation. Its versatility allows for the generation of green hydrogen, produced using renewable energy, which can replace fossil fuels and reduce carbon emissions [16,17]. Hydrogen storage also holds the potential to support energy resilience, as it can be produced and stored locally, reducing the need for extensive energy transmission infrastructure. Such decentralization enhances energy security and minimizes energy losses during transportation. Hydrogen storage is not merely a technical challenge but a linchpin in the transition to a sustainable energy future. Its development and implementation are integral to reducing emissions, increasing energy efficiency, and ensuring energy security. As research and innovation in the field of hydrogen storage technologies continue, we are poised to unlock the full potential of hydrogen as a clean energy resource, paving the way for a greener and more sustainable world [18].

Metal oxides, and particularly certain transition metal hydrides, are receiving attention as potential materials for hydrogen storage. These materials can chemically bond with hydrogen atoms to form metal hydrides [19]. Metal hydrides are solid-state compounds capable of storing and releasing hydrogen under controlled conditions. While some metal oxides show promise for hydrogen storage, they also present various challenges [20]. Hydrogen storage via metal oxides typically involves chemisorption, a chemical reaction that binds hydrogen to the metal atoms within the oxide lattice. Some notable examples include TiO_2_, Fe_2_O_3_, ZrO_2_, NiO, ZnO, Al_2_O_3_, and MgO [21,22]. The advantage of metal hydrides lies in their high theoretical hydrogen storage capacity and the reversibility of the hydrogen absorption and desorption process. Researchers can finetune the properties of these compounds by adjusting their composition and structure, optimizing storage capacity, kinetics, and thermodynamic stability. However, achieving fast hydrogen absorption and desorption kinetics while maintaining thermodynamic stability, suitable operating conditions, and cycling durability remains an ongoing challenge in efforts to harness the full potential of metal oxide-based hydrogen storage systems [23].

Polyaniline (PANI), a conducting polymer, has gained attention as a potential material for hydrogen storage due to its unique properties [24]. As hydrogen storage materials, PANI-based membranes offer the advantages of lightweight and flexible structures. These membranes can reversibly adsorb and desorb hydrogen gas, making them suitable for storing and releasing hydrogen in controlled environments [25]. PANI’s ability to undergo a reversible chemical reaction with hydrogen, forming a stable interaction, contributes to its hydrogen storage potential. Furthermore, the process of hydrogen adsorption and desorption on PANI can be tuned by altering the material’s structure and morphology, offering the possibility of optimizing storage capacity and kinetics. However, challenges remain in achieving high storage capacity and improving the efficiency of the adsorption and desorption processes. Researchers continue to explore and refine the use of PANI membranes as a means of hydrogen storage, aiming to harness their full potential in clean energy applications [26–28].

Chitosan, a biopolymer derived from chitin, has shown promise as a material for hydrogen storage membranes. Its application in hydrogen storage is primarily due to its excellent film-forming properties and high surface area, which can be advantageous for adsorbing and desorbing hydrogen molecules [29,30]. Chitosan-based membranes have the potential to store hydrogen through physisorption, where hydrogen molecules are physically adsorbed onto the surface. While chitosan itself may not possess the highest storage capacity compared to other materials, it offers advantages in terms of safety, cost-effectiveness, and ease of modification. Researchers are exploring ways to enhance the hydrogen storage properties of chitosan membranes through modifications such as the incorporation of nanoparticles or doping with metal ions to improve storage capacity and kinetics. Chitosan-based hydrogen storage membranes hold promise as an environmentally friendly and renewable approach to hydrogen storage and are the subject of ongoing research to optimize their performance for various clean energy applications [31–34].

In the field of advanced materials, innovation often arises from the synergy of different components. The present study utilizes PANI-coated TiO_2_ core-shell nanoparticles embedded within a chitosan membrane as a striking example of this collaborative approach. This amalgamation of components creates a flexible and high-performance material with the capacity to revolutionize diverse fields. From environmental remediation to medical advancements, this composite promises cleaner environments, enhanced healthcare, and sustainable technologies. As research and development efforts continue, the potential for this innovative material to drive positive change across a spectrum of applications is boundless. This composite material can be further assessed for hydrogen adsorption and desorption studies for the purpose of hydrogen storage for fuel-cell applications.

## 2. Materials and methods

The chemicals used in this study were obtained from S.D. Fine Chemicals Pvt. Ltd. (Bengaluru, India). These chemicals included sodium hydroxide (NaOH), sulfuric acid (H_2_SO_4_) with purity of 99%, titanium acetate anhydride (Ti(CH_3_COO)_2_(H_2_O)) with purity of 98.9%, ammonium lauryl sulfate with purity of 99.5%, orthophosphoric acid (H_3_PO_4_) with purity of 99.0%, hydrogen peroxide (H_2_O_2_) of laboratory-reagent grade and molecular weight of 34.01, concentrated hydrochloric acid at a concentration of 35.86%, toluene-4-sulfonic acid monohydrate (C_7_H_8_O_3_S·H_2_O) with molecular weight of 172.20 and purity of 99.4%, ammonium persulfate ((NH_4_)_2_S_2_O_8_) with purity of 99.9%, polystyrene with molecular weight of 350,000 and purity of 99.9%, camphorsulfonic acid (C_10_H_16_O_4_S) with purity of 99.9%, and acetone of analytical grade. These chemicals were used in their original states without any modifications.

### 2.1. Synthesis of porous TiO_2_ nanoparticles

The TiO_2_ nanoparticles were synthesized using the sol-gel technique in the following manner: In a round-bottom flask, a solution was prepared by dissolving 4.5 g of titanium acetate anhydride (Ti(CH_3_COO)_2_(H_2_O)) in 100 mL of ethanol. Another solution was prepared by dissolving 4.5 g of NaOH in 200 mL of ethanol. Initially, a solution of titanium acetate was prepared in a 500-mL beaker while ensuring continuous agitation for 20 min. Subsequently, that solution underwent gradual introduction of the sodium hydroxide solution followed by the addition of hydrogen peroxide (H_2_O_2_) while maintaining continuous agitation at ambient temperature for 3 h. The pH of the solution was carefully maintained within the range of 5.5 to 6.0. To facilitate the sedimentation process of the white colloidal solution, the pH was adjusted using ethanol with a pH value below 8.0 and the solution was allowed to settle for 5 h. The colloidal solution, which exhibited a white appearance, was added to centrifuge tubes and subjected to centrifugation for 20 min at a rotational speed of 15,000 rpm. The liquid portion above the precipitate was discarded and the residual white substance was subsequently washed six times using distilled water. The collected residue underwent a drying process at a temperature of 65 °C for 1 h. Subsequently, it was subjected to annealing at a temperature of 500 °C for 2 h in a muffle furnace equipped with temperature control. Following the annealing process, the resulting residue consisting of TiO_2_ nanoparticles was fragmented into minuscule particles to facilitate their use in the synthesis of the desired nanocomposite [35].

### 2.2. Surface modification of TiO_2_

Sonication was employed to achieve the dispersion of 1 g of TiO_2_ in a solution of 2 M hydrochloric acid (HCl). Subsequently, 1 mL aniline was added to that mixture, followed by agitation for 2 h. A stoichiometric solution of ammonium persulfate, in accordance with the molar ratio of aniline, was employed for the polymerization of aniline under ambient conditions. The PANI-TiO_2_ core-shell matrix solution was subjected to cryogenic conditions by transferring it to a low-temperature environment, specifically a deep freezer, where it remained undisturbed for 24 h. Upon completion of the reaction, a distinct precipitate exhibiting a greenish hue was observed. The precipitated solid was subjected to centrifugation followed by rinsing with a 0.1 M hydrochloric acid (HCl) solution to eliminate any remaining unreacted monomers. Subsequently, a series of aqueous rinses using distilled water were conducted to eliminate any lingering salt residues. To attain a consistent mass, the precipitate underwent a 48-h desiccation process at 50 °C under the influence of a dynamic vacuum. In the previous step, the precipitate also underwent a thorough rinsing utilizing acetone, which effectively eliminated any residual water present within the nanocomposite [36–38].

### 2.3. Membrane preparation

The TiO_2_ was prepared using AEROXIDE hydrophilic fumed TiO_2_ P25 (Evonik, Essen, Germany) with average particle size of 49.5 nm and specific surface area of 50.15 m^2^/g. The TiO_2_-PANI core-shell (with weight percentages of plain chitosan of 0, 2, 4, and 6 wt.%, corresponding to 0, 100, 200, and 300 mg) underwent dispersion in 20 mL of water for 2 h at room temperature through the application of ultrasonication. The core-shell PANI-TiO_2_ nanoparticles were thoroughly dispersed and subsequently combined with a chitosan solution containing 5 wt.% concentration in aqueous medium. The mixture was allowed to react for 48 h. Under anhydrous conditions, the highly viscous suspension was deposited onto a pristine glass substrate [38,39]. For the PANI-TiO_2_-embedded chitosan membrane matrix formulations, the successful creation of self-supporting films with thicknesses ranging from 60 to 65 μm was effortlessly accomplished. The optimal weight percentages of PANI and TiO_2_ were found to balance their synergistic properties, resulting in improved performance compared to individual components or nonoptimized composites. The chitosan and nanoparticles were found to be better amalgamated at 2–6 wt.% with regard to the base matrix of chitosan and we prepared different membrane variants with varying core-shell nanoparticle incorporations on the chitosan membranes. The Table depicts the systematic nomenclature assigned to the synthesized membranes.

## 3. Characterization techniques

The Fourier transform infrared (FTIR) spectra of PANI and the PANI-TiO_2_ nanocomposite were acquired utilizing an IR-Affinity-1 spectrophotometer (Shimadzu, Kyoto, Japan) operating within the wavenumber range of 400–4600 cm^−1^. The generated samples underwent a thorough blending process with potassium bromide (KBr) at a ratio of 1 part sample to 5 parts KBr, resulting in the attainment of a homogeneous paste. Following that procedure, the paste underwent compression via a hydraulic press, resulting in the production of a 10-mm plate. The study primarily focused on the examination of the surface morphology of chitosan and PANI-TiO_2_ core-shell nanocomposite membranes subjected to gold particle deposition via sputtering techniques. This deposition was specifically carried out on a Au substrate. The particle size distribution was ascertained employing a dynamic light scattering technique utilizing a Zetasizer instrument (Malvern PANalytical Ltd., Malvern, UK). The specific surface area was determined using the Brunauer–Emmett–Teller (BET) technique, employing the M3Flex surface characterization analyzer (Micromeritics, Norcross, GA, USA). The electrochemical investigations were carried out using the 660D electrochemical workstation (CH Instruments, Bee Cave, TX, USA), which was equipped with a picoampere booster to enhance the measurement of current and voltage. A series of electrochemical experiments were performed employing a three-electrode setup. The initial operational electrodes were synthesized employing PANI and its nanocomposite, accompanied by a counter electrode composed of a platinum wire and a reference electrode consisting of a saturated calomel electrode. The determination of direct current (DC) conductivity was conducted using a two-probe methodology, employing a 2400 Series Source Meter instrument (Keithley, Cleveland, OH, USA). The cyclic voltammetry (CV) system comprised an electrolysis cell, a potentiostat, a current-to-voltage converter, and a data acquisition system. The electrolysis cell was composed of four discrete constituents, including the working electrode, counter electrode, reference electrode, and electrolytic solution. CV is extensively utilized in the realm of electrochemical investigation owing to its capacity to assess reaction mechanisms utilizing economically viable instrumentation and conducting experiments with temporal efficiency. The experimental procedure involved the utilization of the Interface 1010B potentiostat (Gamry Instruments, Warminster, PA, USA) for the purpose of conducting CV procedures. To assess the operational efficacy of the synthesized nanocomposite membrane, electrochemical impedance spectroscopy (EIS) was employed. EIS allows for the determination of key properties such as ohmic resistance, conductance, and activation energy within synthesized nanocomposite membranes. The specific instrument utilized for this purpose was a Model IM3570 device (Hioki, Nagano, Japan). The investigation focused on evaluating the hydrogen sorption properties of PANI and its nanocomposites while utilizing the PCTPro 2000 sorption apparatus (Hy-Energy, Newark, CA, USA). The software subroutines employed for hydrogen purging cycles were analyzed with the HyDataV2.1 Lab-View tool (Hy-Energy, USA).

## 4. Results and discussion

### 4.1. FTIR spectra

**Figure 1:**
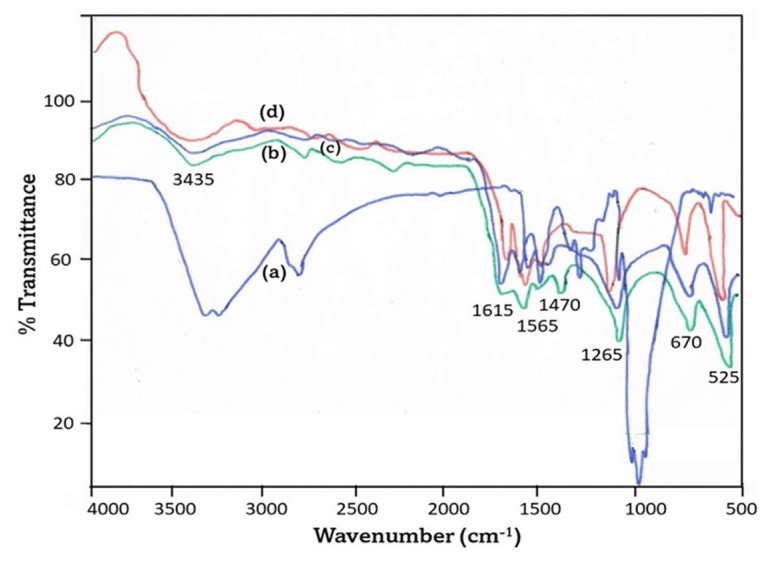
FTIR spectra of chitosan (a), TiO_2_ (b), PANI-coated TiO_2_ core-shell in chitosan nanocomposite membrane (c), and PANI (d).

The FTIR spectra of PANI, TiO_2_, PANI-coated TiO_2_, and chitosan composite thin films are illustrated in Figure 1. The infrared spectrum of chitosan is illustrated in Figure 1a. The strong band observed in the range of 3291–361 cm^−1^ corresponds to the stretching vibrations of N-H and O-H bonds, as well as the presence of intramolecular hydrogen bonding. The absorption bands observed at approximately 2921 and 2877 cm^−1^ can be attributed to the symmetric and asymmetric stretching of the C-H bonds, respectively. The observed bands exhibit specificity toward polysaccharides and can potentially manifest in the spectra of other polysaccharides, including xylan [40], glucans [41], and carrageenans [42]. The spectral peaks observed at approximately 1645 cm^−1^, corresponding to the C=O stretching of amide I, and at 1325 cm^−1^, corresponding to the C-N stretching of amide III, provided evidence for the existence of residual N-acetyl groups. The observed spectral peak at a wavenumber of 1260 cm^−1^ was assigned to the bending vibrations of hydroxyl groups present in the chitosan molecule, as previously reported in the literature [43]. The observed spectral peak at 896 cm^−1^ was attributed to the vibrational motion of the CH group, and more specifically to the bending motion that occurs perpendicular to the plane of the monosaccharide ring.

The FTIR spectra of the pristine titanium dioxide (TiO_2_) sample, as depicted in Figure 1b, exhibited two distinct absorption peaks at wavenumbers 670 and 525 cm^−1^, corresponding to the characteristic vibration band of TiO_2_ [44]. The vibrational mode associated with the O-H group in water molecules is accountable for the broad spectral band detected at a wavenumber of 3434 cm^−1^ [45,46]. The quinoid and benzenoid structures of PANI were spectroscopically characterized by the presence of bands at 1566 and 1469 cm^−1^, respectively. As seen in the Figure 1c, in the case of PANI-coated TiO_2_ in chitosan nanocomposite membranes, it was observed that the aforementioned peaks underwent a shift toward the wavenumbers of 1278 and 1267 cm^−1^, respectively. The observed spectral features at 1486 and 1560 cm^−1^ could be ascribed to the vibrational and stretching modes of the pyrrole ring. The presence of vibrational peaks corresponding to Ti-O-Ti bonds and characteristic vibrational peaks of PANI in the FTIR spectra suggests the occurrence of interactions between TiO_2_ particles within the PANI matrix. Figure 1d represents the PANI peaks.

The FTIR analysis highlighted specific functional groups and how they changed as a result of interactions in the nanocomposite. The FTIR spectra showed interactions between PANI and TiO_2_, with shifting and changing intensity of specific peaks corresponding to PANI’s quinoid and benzenoid structures. In particular, the peaks at about 1565 cm^−1^, associated with quinoid imine (C=N) groups, and about 1470 cm^−1^, corresponding to benzenoid amine (C=C), showed slight shifts or changes in intensity. These changes indicated that TiO_2_ interacts with PANI’s π-conjugated system, possibly through hydrogen bonding or coordination interactions with the lone pairs of nitrogen in PANI. The broad band at 3435 cm^−1^, attributed to N-H stretching vibrations, shifted or broadened, indicating an interaction between PANI’s amine groups and the surface hydroxyls or oxygen atoms of TiO_2_. The incorporation of TiO_2_ alters the electronic environment of PANI, modifying its quinoid and benzenoid structures through a chemical interaction between the PANI functional groups and TiO_2_. The spectral shifts confirmed this. The findings of this study thus demonstrate that the properties of PANI and colloidal TiO_2_ materials are suitable for utilization in electrical and optoelectronic domains.

### 4.2. XRD pattern

**Figure 2:**
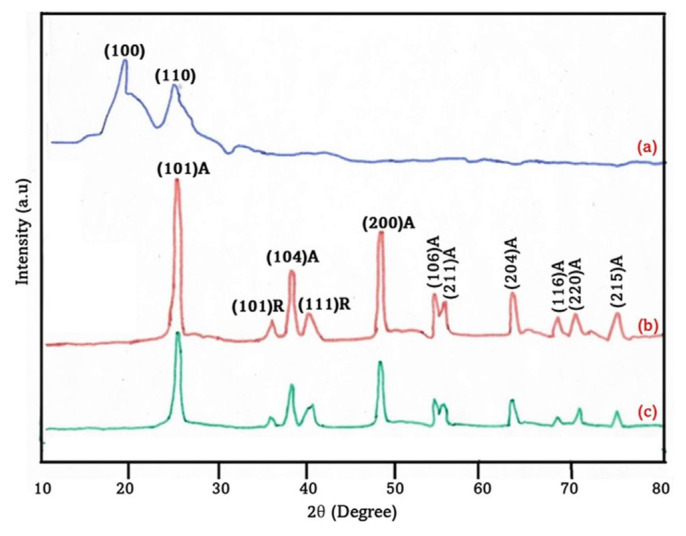
XRD patterns of a standard sample (a), pristine TiO_2_ (b), and TiO_2_/PANI core-shell nanoparticles (1:1) (c).

Figure 2 presents X-ray diffraction (XRD) patterns elucidating the structural characteristics of PANI, TiO_2_, and nanocomposite thin films comprising TiO_2_ and PANI. The XRD pattern obtained from the pure PANI sample, as depicted in Figure 2a, exhibited broad peaks that could be attributed to the crystallographic (100) and (110) planes. This observation is consistent with the expected values reported in the standard reference of JCPDS No. 53-1718. Figures 2b and 2c present the XRD patterns of pristine TiO_2_ and the composite TiO_2_/PANI material, respectively. The observed spectra exhibited distinct and sharp peaks, suggesting the crystalline nature of the samples. The observed diffraction peaks can be ascribed to a tetragonal crystal structure comprising a combination of anatase and rutile phases. The calculated lattice parameters, specifically a = 3.6983 Å and c = 9.4823 Å, were in good agreement with the relevant JCPDS data (Card No. 78-1285). Upon comparison with TiO_2_, it was observed that the diffraction peaks of TiO_2_/PANI exhibited broadening and a decrease in intensity. This suggested that the inclusion of weakly crystalline PANI and the subsequent reduction in the volume fraction of TiO_2_ sequentially led to a diminishment of the TiO_2_ diffraction peaks within the composites. Moreover, the phenomenon of scattering arising from the presence of PANI chains at specific interplanar distances resulted in a decrease in the growth of individual grains in the unmodified TiO_2_ material. Upon the adsorption of PANI chains onto the surface of the TiO_2_ nanoparticles, it was observed that the crystallinity of the PANI was compromised as a result of the constraining influence exerted by the TiO_2_ nanoparticles. By employing Scherrer’s formula [47], we successfully ascertained the average grain size of the pristine TiO_2_ and TiO_2_/PANI nanocomposite films to be 32 and 25 nm, respectively.

The TiO_2_/PANI nanocomposite had lattice parameters (a = 3.6983 Å and c = 9.4823 Å) consistent with anatase-phase TiO_2_ and these values could be compared to standard anatase TiO_2_ lattice parameters from the literature (a ≈ 3.785 Å and c ≈ 9.514 Å). The slight deviation suggests lattice distortion due to the interaction between TiO_2_ and PANI. PANI incorporation may modify the crystalline structure. This comparison demonstrates the effect of nanocomposite formation on lattice parameters.

### 4.3. Scanning electron microscopy (SEM) analysis

**Figure 3:**
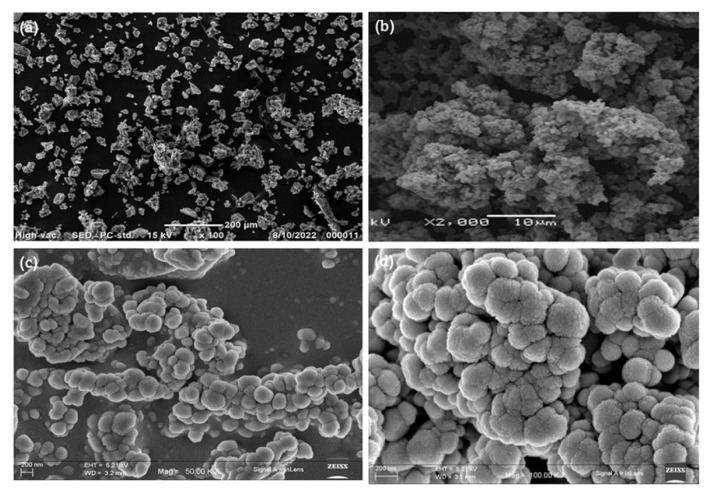
SEM images of **(a)** TiO_2_, **(b)** PANI, **(c)** 4 wt.% PANI-TiO_2_ nanocomposite, and **(d)** 6 wt.% PANI-TiO_2_ nanocomposite.

Due to the favorable surface-to-volume ratio, the synthesized metal oxide and the polymer (PANI) impregnated with the metal oxide exhibited notably enhanced hydrogen storage capabilities compared to bulk materials. As depicted in Figure 3a, the titanium precursor within the soft template array facilitated the formation of highly defined TiO_2_ nanoparticles. Additionally, these nanoparticles exhibited grain sizes of approximately 60 nm. Moreover, when the concentration of TiO_2_ nanoparticles was below 1% in an aniline solution during the process of in situ polymerization, the nanoparticles underwent complete immersion or coating by the PANI without any agglomeration of the nanoparticles, as depicted in Figure 3b. The meticulously regulated in situ polymerization procedure employing an anionic surfactant facilitated the strategic development of the desired TiO_2_-doped PANI nanocomposite [48–50].

The SEM images showed the coating effect of PANI on TiO_2_ nanoparticles and their agglomeration state. The presence of PANI may have led to a more uniform texture across particle surfaces. Smoother or less defined edges on particles may indicate a PANI conformal coating. Figures 3a and 3b depict particles that are loosely packed, indicating some degree of agglomeration. However, in Figures 3c and 3d, individual particles or smaller clusters are more distinguishable, implying that PANI may have reduced the excessive aggregation. PANI dispersed the TiO_2_ particles by creating a polymeric barrier that reduced agglomeration due to van der Waals forces and other interactions. Coating TiO_2_ with PANI reduced the interparticle interactions and improved the dispersion. Higher magnification as seen in Figures 3c and 3d could reveal gaps between clusters or particles, further confirming this effect.

### 4.4. Particle size analysis

**Figure 4:**
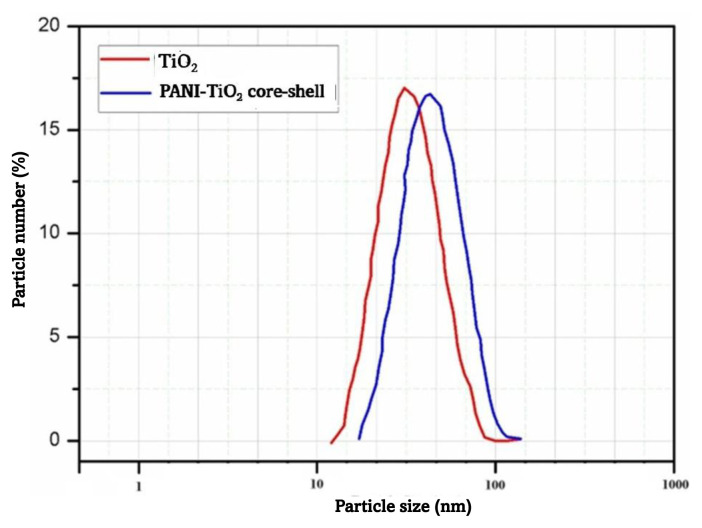
Histograms of particle size distribution of TiO_2_ and PANI-coated TiO_2_ core-shell nanoparticles.

The graphical representation in Figure 4 illustrates the spatial arrangement of TiO_2_ particles and the PANI-coated TiO_2_ core-shell nanoparticles. The temporal parameter employed for dispersion was held constant. The TiO_2_ nanoparticles exhibited mean respective particle dimensions of 49.5 and 60.9 nm. The maximum particle size of the TiO_2_ sample was observed to be approximately 100 nm. The PANI-coated TiO_2_ core-shell nanoparticles exhibited a mean particle size of 60.9 nm, with maximum particle size reaching 120 nm. The aniline underwent polymerization and was subsequently deposited onto the TiO_2_ surface, leading to the formation of core-shell nanoparticles. These nanoparticles exhibited mean thickness of approximately 11.4 nm, as determined by the difference between the mean values of 60.9 nm and 49.5 nm.

### 4.5. Surface area and pore-size analysis

**Figure 5:**
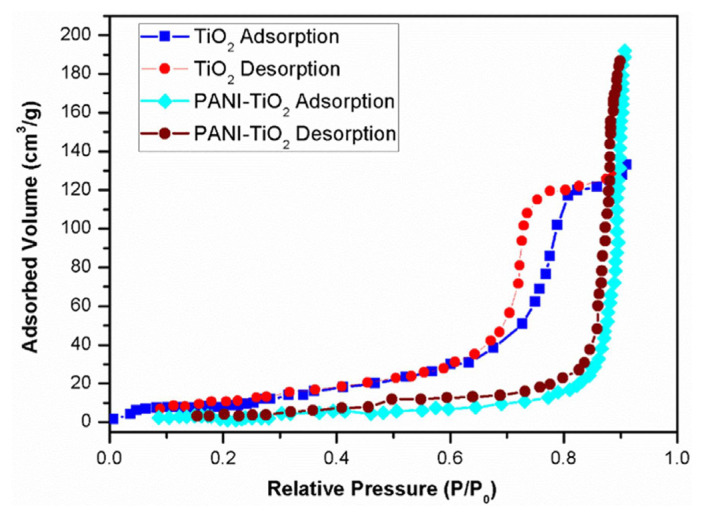
BET analysis of TiO_2_ and PANI-coated TiO_2_ core-shell nanoparticles.

Hysteresis curves are illustrated in Figure 5. In that figure, the curve with the nature of a type IV isotherm is typical of mesoporous materials and indicates capillary condensation within the pores at higher relative pressure. The type H1 hysteresis loop reflects a uniform pore size distribution as well as cylindrical or slit-like pores. The relationships among surface area, porosity, and hydrogen storage are as follows: higher surface area means more adsorption sites for hydrogen molecules, whereas well-defined mesoporosity increases the storage capacity by facilitating gas diffusion and optimizing pore-filling mechanisms. The combination of PANI’s conductivity and TiO_2_’s porous structure in the PANI-TiO_2_ nanocomposites improved the hydrogen adsorption and storage efficiency.

**Figure 6:**
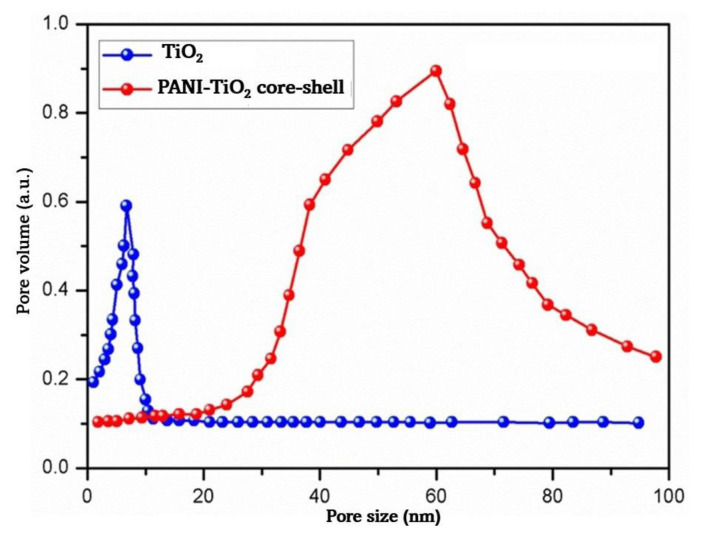
Pore size distribution of TiO_2_ and PANI-coated TiO_2_ core-shell nanoparticles.

The specific surface area and pore-size distribution of both synthesized TiO_2_ nanoparticles and PANI-coated TiO_2_ core-shell nanoparticles were determined through the utilization of nitrogen gas adsorption [51]. A graphical representation was prepared to illustrate the presence of mesoporosity in anatase TiO_2_. The determination of the specific surface area of TiO_2_ was accomplished through the utilization of the widely accepted multipoint BET method, yielding a value of 20.28 m^2^/g. Remarkably, this value was found to be merely one-third the specific surface area exhibited by the PANI-coated TiO_2_ nanoparticles, which amounted to an impressive 48.12 m^2^/g. As depicted in Figure 6, the synthesized TiO_2_ nanoparticles exhibited a narrow distribution of pore sizes, with a mean value of 61.28 nm for the PANI-coated TiO_2_ nanoparticles and 17.89 nm for the uncoated TiO_2_ nanoparticles. The hydrogen storage performance of the TiO_2_ nanoparticles was significantly influenced by the specific porosity and particle and pore size, as well as the charge transport properties [52]. Nanoparticles of reduced dimensions exhibit an augmented surface area, albeit accompanied by diminished electron diffusion length. Conversely, nanoparticles of increased dimensions had an expanded surface area together with elongated electron diffusion length [53].

### 4.6. DC conductivity

**Figure 7:**
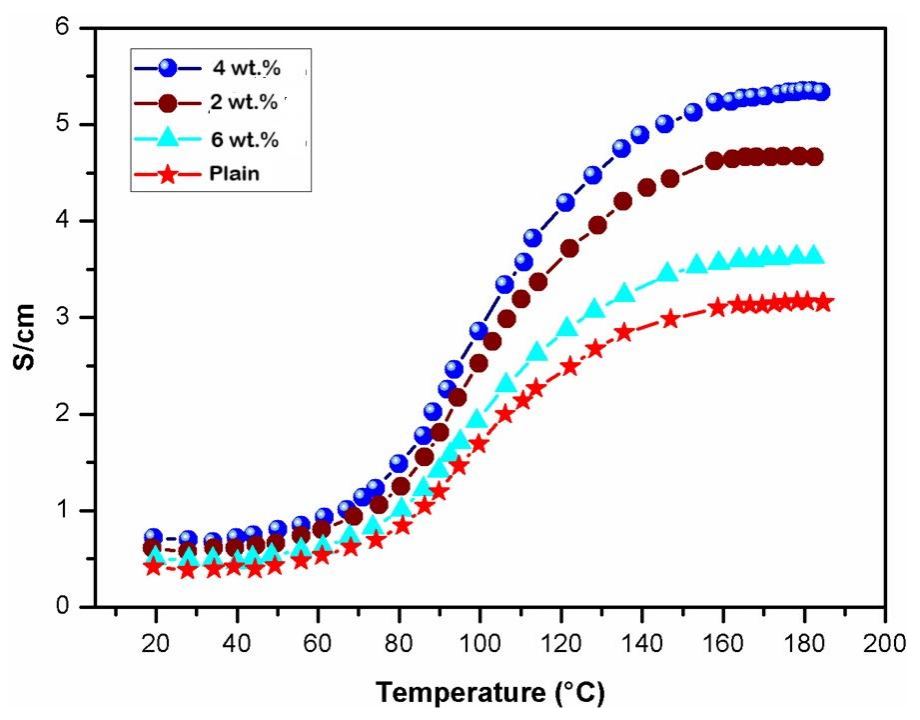
DC conductivity of PANI and PANI-TiO_2_ nanocomposites.

Figure 7 illustrates the DC conductivity of a pristine chitosan membrane and PANI-coated TiO_2_ core-shell chitosan nanocomposites, where temperature serves as the independent variable. The observed conductive properties of chitosan and its nanocomposites exhibited progressive growth in response to varying temperatures, ranging from 25 to 200 °C. This phenomenon can be ascribed to the amplification of charge carriers occurring within the system. With an increase in temperature, there is a corresponding elevation in DC conductivity, leading to a decrease in the activation energy (E_a_). This can be attributed to perturbation in the Fermi level within the PANI-doped TiO_2_ core-shell chitosan nanocomposites. The conductivity of the nanocomposites displayed characteristic semiconductor behavior, reflecting a discernible dependence on temperature. This phenomenon can be effectively captured by employing the one-dimensional variable-range hopping (1D-VRH) model, as originally formulated by Mott and Davis:


$$\rho (T)=\rho_{0}\;exp[{\left(T0/T\right)}^{1/p}]$$

The preexponential factor, denoted as *ρ*_0_, is solely dependent on the reduced temperature. T represents the thermodynamic parameter reflecting the critical temperature at which the phenomenon of molecular hopping occurs, while T_0_ symbolizes the characteristic activation energy factor associated with the aforementioned process. It has been established in the literature that a DC conductivity value of zero signifies the prevalence of conduction primarily in states that exhibit significant spatial dispersion [54,55]. A value of zero signifies a broad spectrum of localized states, and the process of conduction takes place through the mechanism of charge carrier hopping from the valence bands to the conduction bands. Due to the diverse assortment of localized states observed within the nanocomposites of the present study, conductivity measurements performed for the chitosan membrane in its pure form and the membrane containing 4 wt.% nanocomposite membranes yielded values of approximately 5–6 S/cm. This suggests that the phenomenon of conduction takes place through a mechanism known as hopping. Based on the empirical findings, it can be inferred that the phenomenon of hopping serves as the fundamental mechanism accountable for the observed enhancement in the conductivity of the nanocomposite material [56,57]. The generation of polarons and bipolarons additionally offers deeper insights into the intricacies of the conduction mechanism. The presence of polarons and bipolarons exerts a significant influence on the charge injection dynamics within the transport characteristics of PANI nanocomposites [58]. These self-localized defects manifest as distortions in the PANI backbone and quantum states residing within the energy gap due to robust lattice coupling.

The conductivity of the PANI/TiO_2_ nanocomposites increases with temperature due to thermally activated charge transport. This behavior is explained by a hopping mechanism in which charge carriers (polarons or bipolarons) overcome localized potential barriers using thermal energy. The hopping mechanism is closely linked to temperature, as higher temperatures provide the energy required for carriers to hop between localized states. As a result, conductivity increases exponentially with temperature, which is consistent with Mott variable range hopping or Arrhenius-like behavior. To provide a more comprehensive explanation, theoretical models could be applied to the conductivity–temperature relationship for an exploration of how the TiO_2_ contents affect carrier mobility and hopping distances.

The temperature-dependent conductivity of the core-shell nanocomposite membranes reflected a triphasic response. The first phase of the temperature range, from 20 to 80 °C, was almost constant, which may have been due to the fact that there was not enough energy for the polarons and bipolarons to move from a lower energy state to a higher energy state. The second stage showed an incremental increase in conductivity between 80 and 120 °C, which can be attributed to the system’s increased electron charge mobility. The third phase exhibited a sharp increase in conductivity from 120 to 190 °C, mainly caused by the migration of bipolarons and polarons from an excited state to ground state. Semiconductors made of PANI exhibit such behavior. It was previously reported that such nanocomposites with a 4 wt.% membrane had maximum conductivity of 5.7 S/cm [59].

### 4.7. Cyclic voltammetry

A functional electrode was synthesized utilizing a PANI-coated polyester substrate. The conducting substance employed in the experiment consisted of chitosan and its nanocomposites, whereas the utilized support material was polystyrene. In isolation, PANI fails to produce a coating possessing sufficient mechanical characteristics. Consequently, polystyrene served as the substrate material in the synthesis of a composite containing conductive PANI. The coating solution was synthesized and the deposition process was carried out. Initially, 1 g of PANI and 1.28 g of camphorsulfonic acid (C_10_H_16_O_4_S) (CSA) were solubilized in 20 mL of chloroform. Subsequently, 1.00 g of polystyrene was introduced into the solution. The coating solution was synthesized via magnetic agitation for 24 h. A solution of PANI and CSA in chloroform was prepared, followed by the addition of a solution of polystyrene. The final product was employed to synthesize a polymer solution for the purpose of coating. The PANI underwent coupling with CSA, resulting in the formation of a PANI-CSA salt. The molar ratio employed was 2:1, with the PANI quantity determined based on the aniline monomeric unit. At this stoichiometric proportion, the PANI-CSA salt exhibited favorable solubility in chloroform. Due to the adequate conductivity of this blend, the weight ratio of PANI to polystyrene employed in this investigation was 1:1 [60–62].

### 4.8. Cyclic voltammetry study

**Figure 8:**
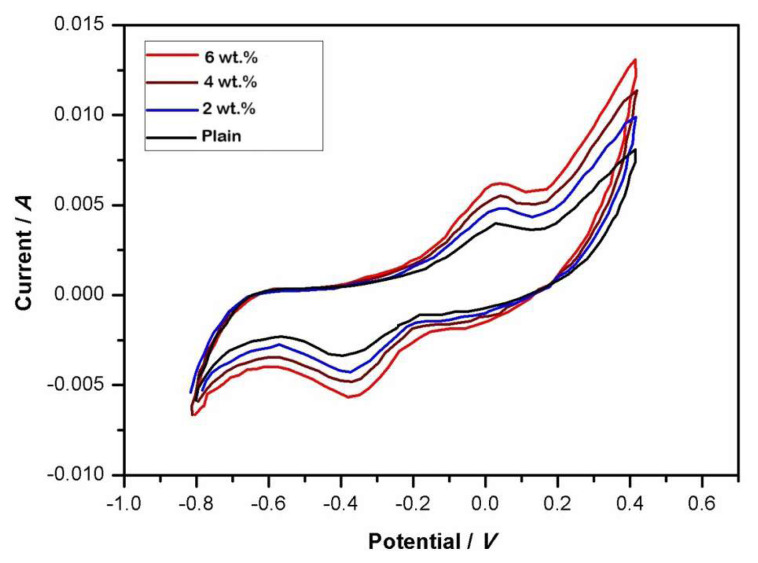
Cyclic voltammograms of PANI and PANI-TiO_2_ nanocomposites.

The CV profiles displayed in Figure 8 showcase the electrochemical behavior of both plain chitosan and PANI-coated TiO_2_ core-shell nanocomposite membranes. These profiles exhibit a distinct rectangular morphology, which is indicative of the presence of electrochemical pseudocapacitive behavior. The CV curve of chitosan, in contrast, exhibits the typical CV behavior frequently observed for chitosan. The redox peaks observed at 0.94 V and 0.25 V can be attributed to specific electrochemical processes associated with chitosan. The presence of the two peaks can be ascribed to the redox transition of PANI, wherein it undergoes a shift from a semiconducting state to a conducting state. More precisely, this transition involves the conversion of the polaronic emeraldine form to the emeraldine-pernigraniline state [63].

The doping/dedoping process is responsible for PANI’s transition from semiconductor to conductor state in the CV profiles. During the forward scan, PANI oxidized, resulting in the formation of polarons and bipolarons, the charge carriers responsible for conductivity. This redox process enhanced electronic delocalization, causing the PANI to conduct. On the reverse scan, dedoping occurred, restoring the PANI to its semiconducting state. The effects on material properties included increased conductivity and charge storage capacity in the conducting state, as well as changes in electrochemical activity and electron transport dynamics. A more detailed explanation could center on correlating the observed current response to specific oxidation states of PANI and related structural changes.

### 4.9. Electrical impedance spectroscopic studies

**Figure 9:**
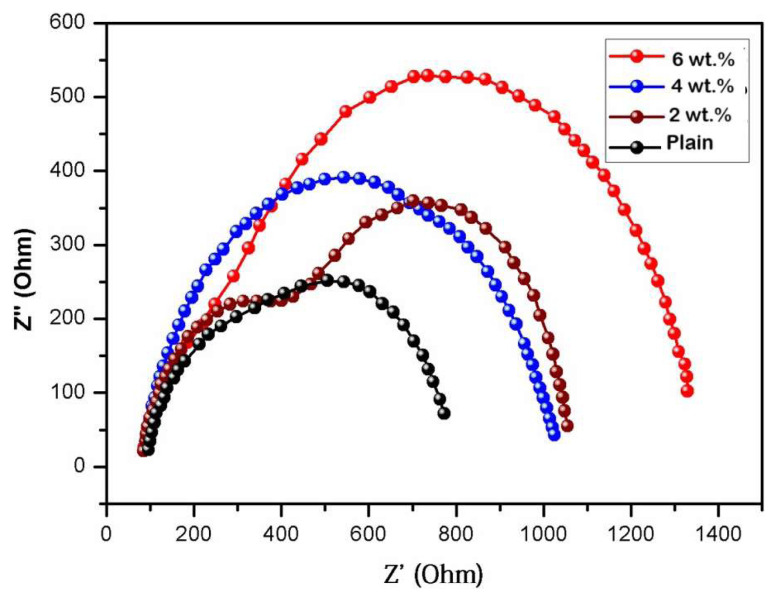
Impedance plots (Nyquist curves) for plain chitosan and 2 wt.%, 4 wt.%, and 6 wt.% membranes.

Upon careful examination of Figure 9, it becomes apparent that the semicircle exhibited by the core-shell nanocomposite membranes surpasses that of the plain chitosan membrane in terms of size. This observation strongly implies that our material possesses superior conductive characteristics. Furthermore, it is worth noting that the optimal resistance to charge transfer, denoted as R_ct_, was achieved upon subjecting the film to potential of 30 V. This outcome aligns with the estimations derived from the Tafel curves. The observed findings suggest that the enhanced electrical conductivity of the membranes can be attributed to a compact and enduring arrangement, along with a uniform surface exhibiting minimal porosity and fissures [64,65].

### 4.10. Hydrogen sorption measurements

Volumetric hydrogen sorption investigations played a pivotal role in elucidating the hydrogen storage characteristics of the plain chitosan and PANI-coated TiO_2_ nanocomposite membranes. At 60 °C, the process of hydrogen absorption was carried out in a precalibrated reservoir under the influence of high pressure of 80 bar. Isothermal volumetric analyses were conducted using the PCTPro 2000 sorption instrument (Hy-Energy, USA). This apparatus is completely automated, featuring a 170-bar pressure regulator and an integrated PID-controlled pressure regulator. The hydrogen purging cycles, leak testing, and PCT software subroutines were analyzed utilizing the HyData V2.1 Lab-View application [66,67].

The plain chitosan and its nanocomposite membranes underwent meticulous drying prior to their transfer to a glovebox, where they were placed under an atmosphere of inert nitrogen gas. Subsequently, they were transported to a high-pressure hydrogen reactor. The hermetically sealed reactor was connected to a high-pressure volumetric setup with hydrogen to examine the sorption capabilities.

**Figure 10:**
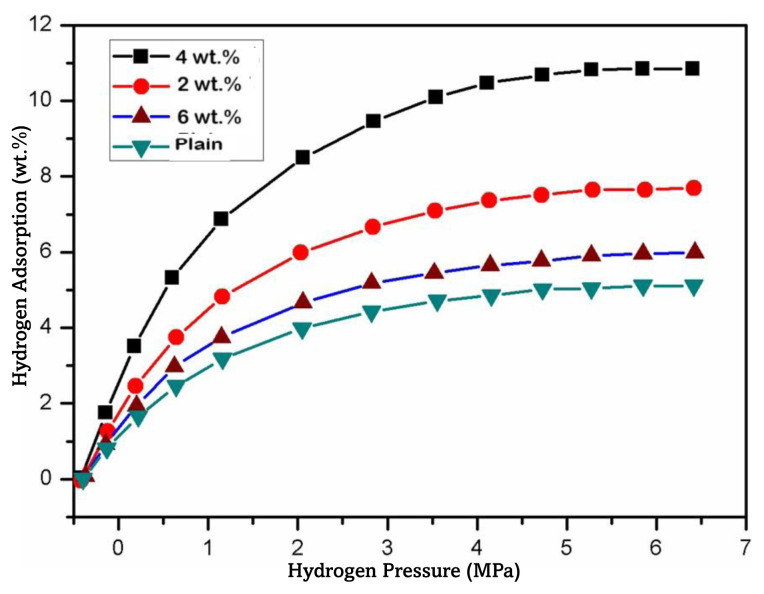
Hydrogen adsorption of PANI and PANI-TiO_2_ nanocomposites.

Figure 10 exhibits the fluctuation in hydrogen uptake under standard ambient conditions as a consequence of the exerted pressure. It was observed that an increase in pressure correlated with an enhanced capacity for hydrogen adsorption. The plain chitosan membrane exhibited notable hydrogen adsorption capacity of approximately 4.5 wt.% at room temperature. However, when the temperature rose to 60 °C, this capacity underwent a twofold increase, reaching approximately 7.4 wt.%. In juxtaposition, a concentration of 4 wt.% nanocomposite membrane had a notable absorption capacity of 10.5 wt.% in comparison to 2 wt.% and 6 wt.% nanocomposite membranes, which may possibly be attributed to their distinctive architecture and morphology, suggesting the nature of the material and hinting at the involvement of nitrogen (N) molecules in PANI as hydrogen bond donors. Furthermore, the presence of numerous hydrogen bond receptor sites within the molecular structure is implied [68]. Hydrogen undergoes adsorption through two distinct mechanisms. The backbones of the plain chitosan and PANI present in the membrane constituted a dual receptor system within the conductive groups, wherein the receptor count escalated in proportion to the quinoid diimine units (N=B=N). This led to a heightened electronic transition, facilitating interaction with hydrogen atoms. During the subsequent phase, the physisorption of H_2_ molecules (referred to as H_2_* species) occurred. This process involved the heterolytic cleavage of the H-H bond, resulting in the formation of a hydride species that bound to the Ti site. Additionally, a proton attached itself to an oxygen atom, leading to the creation of the (H+-H) species. Subsequently, the hydrogen (H) atom residing on the titanium site (Ti^4+^ → Ti^3+^) underwent translocation to the adjacent oxygen (O) site, leading to uniform dissociation and the formation of (H+-H^+^) 2O-H hydroxyl entities. This process was subsequently accompanied by a bielectronic transfer to reduce the surface titanium sites [69].

**Figure 11:**
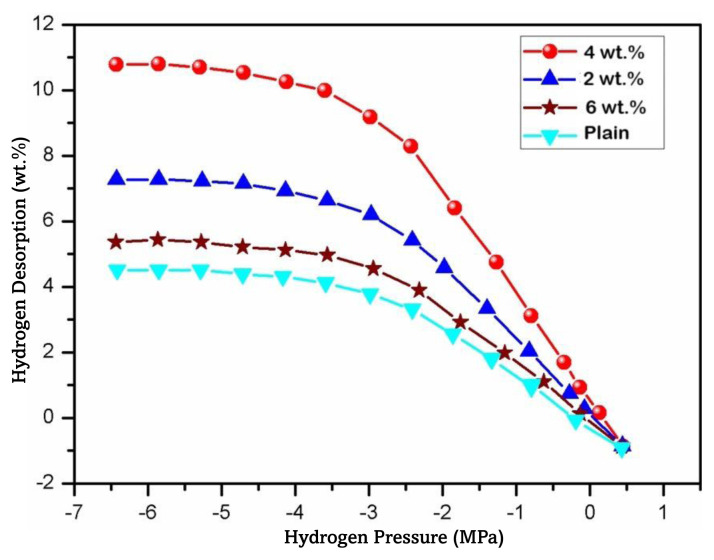
Hydrogen desorption of PANI and PANI-TiO_2_ nanocomposites at 60 °C.

Figure 11 showcases the hydrogen desorption of plain chitosan and PANI-coated TiO_2_ embedded chitosan nanocomposite membranes at 60 °C, with the applied pressure serving as the variable. It was observed that elevating the temperature from ambient conditions to 60 °C resulted in a rise in hydrogen desorption, transitioning from content of 10.4 wt.% to complete absence. Nevertheless, it is imperative to acknowledge that the desorption phenomenon was notably slow under ambient conditions, potentially attributable to the robust N=H interaction inherent in the PANI backbone molecules. Under ambient conditions, the desorption of hydrogen took place at 78% and 86% for the chitosan and PANI-coated TiO_2_ embedded chitosan nanocomposite membranes, respectively [70].

**Figure 12:**
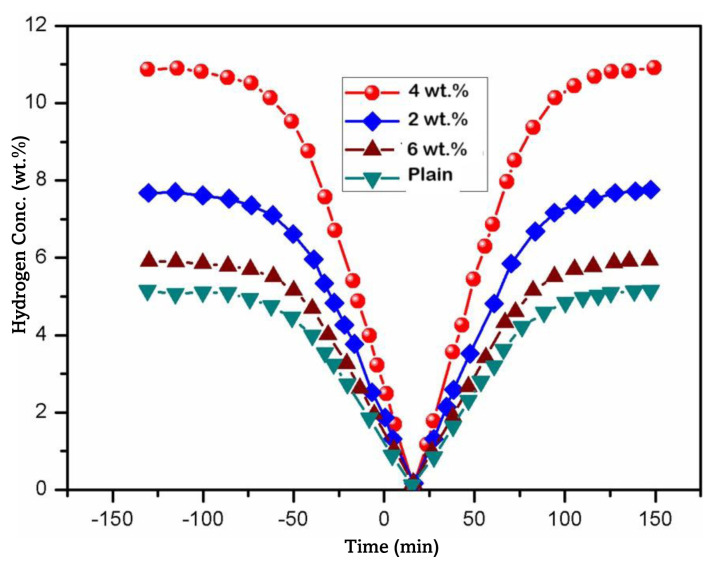
Hydrogen absorption/desorption profiles as a function of time.

As illustrated in Figure 12, the initial hydrogen absorption of the plain chitosan and PANI-coated TiO_2_ embedded chitosan nanocomposite membranes occurred under ambient conditions and was achieved through in situ polymerization in an ice bath over a specific time interval. The nanocomposite membranes exhibited rapid attainment of saturation in less than 80 min, specifically achieving 88% of its maximum absorption capacity, corresponding to 10.5 wt.%. Moreover, upon elevating the temperature to 60 °C under pressure of 1 bar, the prepared nanocomposite membranes (2, 4, and 6 wt.%) exhibited absorption capacity of approximately 2–4 wt.% for hydrogen at the optimum temperature. Nevertheless, the desorption of hydrogen gas required a duration of 140 min, a phenomenon that can be ascribed to the robust N=H interaction exhibited by the backbone molecules present in the PANI and the hygroscopic characteristics of the TiO_2_ nanoparticles [71]. Analysis of the plain chitosan was conducted at ambient temperature to ascertain the efficacy of hydrogen sorption. After 80 min, the application of variable pressure induced the formation of an efficacious hydride at approximately 6 bar. Having a linear region characterized by a concentration of 4.5 wt.%, the hydrogen uptake underwent an exponential decrease until reaching a concentration of 90 wt.%. In the case of the 4 wt.% nanocomposite membrane, it was observed that the overall desorption capacity experienced a decrease, reaching a value of 96% [72]. The obtained results were compared with those of a recent publication and the values were found to be comparable [73].

## 5. Conclusion

Chitosan and its nanocomposite membranes made from porous metal oxides have promising properties as hydrogen storage materials. In this study, chitosan and PANI-coated TiO_2_ nanocomposite chitosan membranes were synthesized. FTIR spectroscopy and XRD were used to characterize the chitosan nanocomposite membranes for structural investigation. The FTIR spectra showed the typical benzenoid ring peak associated with C-N stretching and the quinoid ring peak associated with C-H bending motion, as well as metal oxide peaks. These findings support the successful formation of TiO_2_ within the PANI nanocomposites. The XRD analysis revealed that the PANI and TiO_2_ were semicrystalline, indicating the presence of a rutile phase with a tetragonal nanostructure within the nanocomposites. The surface morphology was investigated using SEM, which confirmed the formation of a fibrous architecture incorporating TiO_2_ nanoparticles. DC conductivity studies revealed trinary conductivity stages typical of a semiconductor. The highest (D) conductivity observed among all nanocomposites was 5.7 S/cm for the nanocomposite composed of 4 wt.% PANI-coated TiO_2_ core-shell embedded chitosan membrane. The properties of the chitosan and its nanocomposite derivatives were investigated using CV, a technique that involved the detection of redox peaks at 0.94 V and 0.24 V. The occurrence of those two peaks can be attributed to the redox transition of the PANI from a semiconducting to a conducting state. The hydrogen absorption capacity was approximately 4.5 wt.% at room temperature but more than doubled to approximately 7.4 wt.% at 60 °C. Compared to the other nanocomposite membranes, the 4 wt.% PANI-TiO_2_ embedded chitosan nanocomposite membrane had significantly higher absorption capacity of 10.5 wt.% and lower desorption capacity, resulting in an overall value of 96%, indicating its intriguing potential for use in hydrogen storage applications.

## Figures and Tables

**Table:** Thicknesses and names of membranes.

No.	Membrane	Thickness (μm)	Name
1	Plain chitosan	60	Plain
2	2 wt.% PANI-TiO_2_ embedded in chitosan	63	2 wt.%
3	4 wt.% PANI-TiO_2_ embedded in chitosan	65	4 wt.%
4	6 wt.% PANI-TiO_2_ embedded in chitosan	65	6 wt.%

## Data Availability

The data presented in this study are available on request from the corresponding author.

## References

[1] Apostolou D Xydis GA literature review on hydrogen refuelling stations and infrastructure. Current status and future prospects Renewable and Sustainable Energy Reviews 2019 113 109 292 10.1016/j.rser.2019.109292

[2] Dincer I Acar C Smart energy solutions with hydrogen options International Journal of Hydrogen Energy 2018 43 18 8579 8599 10.1016/j.ijhydene.2018.03.120

[3] Khan N Kalair E Abas N Kalair AR Kalair A Energy transition from molecules to atoms and photons Engineering Science and Technology an International Journal 2019 22 1 185 214 10.1016/j.jestch.2018.05.002

[4] To WM Lee PKC Energy consumption and economic development in Hong Kong, China Energies 2017 10 11 1883 10.3390/en10111883

[5] Mohsin M Kamran HW Nawaz MA Hussain MS Dahri AS Assessing the impact of transition from nonrenewable to renewable energy consumption on economic growth-environmental nexus from developing Asian economies Journal of Environmental Management 2021 284 111 999 10.1016/j.jenvman.2021.111999 33556829

[6] Sevilla M Fuertes AB Mokaya R Preparation and hydrogen storage capacity of highly porous activated carbon materials derived from polythiophene International Journal of Hydrogen Energy 2011 36 24 15658 15663 10.1016/j.ijhydene.2011.09.032

[7] Chen Y Cao X Zhu H Liu Y Preparation of a porous carbon from ferrocene-loaded polyaniline and its use in hydrogen adsorption International Journal of Hydrogen Energy 2012 37 9 7629 7637 10.1016/j.ijhydene.2011.09.107

[8] Yan H Zhang W Kang J Yuan T The necessity and feasibility of hydrogen storage for large-scale, long-term energy storage in the new power system in china Energies 2023 16 13 4837 10.3390/en16134837

[9] Shen J Wang Y Cheng C Li XF Miao SM Research status and prospect of generation scheduling for hydropower-wind-solar energy complementary system Proc CSEE 2022 42 3871 3885

[10] Gielen D Boshell F Saygin D Bazilian MD Wagner N The role of renewable energy in the global energy transformation Energy Strategy Reviews 2019 24 38 50 10.1016/j.esr.2019.01.006

[11] Li Z Chen SY Dong W Liu P Du E Low carbon transition pathway of power sector under carbon emission constraints Proc CSEE 2021 41 12 3987 4001

[12] Hao X Wang H Lin Z Ouyang M Seasonal effects on electric vehicle energy consumption and driving range: A case study on personal, taxi, and ridesharing vehicles Journal of Cleaner Production 2020 249 119 403 10.1016/j.jclepro.2019.119403

[13] Guo Y Ming B Huang Q Wang Y Zheng X Risk-averse day-ahead generation scheduling of hydro-wind-photovoltaic complementary systems considering the steady requirement of power delivery Applied Energy 2022 309 118467 10.1016/j.apenergy.2021.118467

[14] Zhang X Ma G Huang W Chen S Zhang S Short-term optimal operation of a wind-PV-hydro complementary installation: Yalong River, Sichuan Province, China Energies 2018 11 4 868 10.3390/en11040868

[15] Li M Chen G Dong C Liang Z Wang W Research on power balance of high proportion renewable energy system Power System Technology 2019 43 11 3979 3986

[16] Mohan M Sharma VK Kumar EA Gayathri V Hydrogen storage in carbon materials-A review Energy Storage 2019 1 2 e35 10.1002/est2.35

[17] Zivar D Kumar S Foroozesh J Underground hydrogen storage: A comprehensive review International Journal of Hydrogen Energy 2021 46 45 23436 23462 10.1016/j.ijhydene.2020.08.138

[18] Tarkowski R Uliasz-Misiak B Towards underground hydrogen storage: A review of barriers Renewable and Sustainable Energy Reviews 2022 162 112451 10.1016/j.rser.2022.112451

[19] Chen TW Chen SM Anushya G Kannan R Veerakumar P Metal-Oxides-and Metal-Oxyhydroxides-Based Nanocomposites for Water Splitting: An Overview Nanomaterials 2023 13 13 2012 10.3390/nano13132012 37446527 PMC10343756

[20] Jangir M Jain IP Mirabile Gattia D Effect of Ti-Based Additives on the Hydrogen Storage Properties of MgH_2_: A Review Hydrogen 2023 4 3 523 541 10.3390/hydrogen403003

[21] Tian Z Wang Z Yao P Xia C Yang T Hydrogen storage behaviors of magnesium hydride catalyzed by transition metal carbides International Journal of Hydrogen Energy 2021 46 80 40203 40216 10.1016/j.ijhydene.2021.09.212

[22] Sazelee N Md Din MF Ismail M Rather SU Bamufleh HS Effect of LaCoO_3_ synthesized via solid-state method on the hydrogen storage properties of MgH_2_ Materials (Basel) 2023 16 6 2449 10.3390/ma16062449 36984329 PMC10057918

[23] Chen G Zhang Y Cheng H Zhu Y Li L Effects of two-dimension MXene Ti3C2 on hydrogen storage performances of MgH_2_-LiAlH_4_ composite Chemical Physics 2019 522 178 187 10.1016/j.chemphys.2019.03.001

[24] Mahato N Jang H Dhyani A Cho S Recent progress in conducting polymers for hydrogen storage and fuel cell applications Polymers (Basel) 2020 12 11 2480 10.3390/polym12112480 33114547 PMC7693427

[25] Patil M Mathad SN Patil AY Arshad MN Alorfi HS Synthesis and Characterization of Microwave-Assisted Copolymer Membranes of Poly (vinyl alcohol)-g-starch-methacrylate and Their Evaluation for Gas Transport Properties Polymers (Basel) 2022 14 2 350 10.3390/polym14020350 35054755 PMC8779399

[26] Attia NF Geckeler KE Polyaniline-polypyrrole composites with enhanced hydrogen storage capacities Macromolecular Rapid Communications 2013 34 11 931 937 10.1002/marc.201300060 23625749

[27] Rahy A Rguig T Cho SJ Bunker CE Yang DJ Polar solvent soluble and hydrogen absorbing polyaniline nanofibers Synthetic Metals 2011 161 3–4 280 284 10.1016/j.synthmet.2010.11.036

[28] Nazri GA Hills B GM Global Technology Operations 2008

[29] Modau L Sigwadi R Mokrani T Nemavhola F Chitosan membranes for direct methanol fuel cell applications Membranes (Basel) 2023 13 10 838 10.3390/membranes13100838 37888010 PMC10608347

[30] Selim A Szijjártó GP Tompos A Insights into the Influence of Different Pre-Treatments on Physicochemical Properties of Nafion XL Membrane and Fuel Cell Performance Polymers (Basel) 2022 14 16 3385 10.3390/polym14163385 36015643 PMC9414504

[31] Sherazi TA Guiver MD Kingston D Ahmad S Kashmiri MA Radiation-grafted membranes based on polyethylene for direct methanol fuel cells Journal of Power Sources 2010 195 1 21 29 10.1016/j.jpowsour.2009.07.021

[32] Hwang S Lee H Jeong YG Choi C Hwang I Polymer electrolyte membranes containing functionalized organic/inorganic composite for polymer electrolyte membrane fuel cell applications International Journal of Molecular Sciences 2022 23 22 14252 10.3390/ijms232214252 36430726 PMC9694323

[33] Chowdury MSK Cho YJ Park SB Park Y Functionalized graphene oxide membranes as electrolytes Journal of the Electrochemical Society 2023 170 3 33503 10.1149/1945-7111/acc35e

[34] Adiyar SR Satriyatama A Azjuba AN Sari N An overview of synthetic polymer-based membrane modified with chitosan for direct methanol fuel cell application IOP Conference Series: Materials Science and Engineering 2021 IOP Publishing 1 2002 10.1088/1757-899X/1143/1/012002

[35] Li Y White T Lim SH Structure control and its influence on photoactivity and phase transformation of TiO Reviews on Advanced Materials Science 2003 5 211 215

[36] Balikile RD Roy AS Nagaraju SC Ramgopal G Conductivity properties of hollow ZnFe_2_ O_4_ nanofibers doped polyaniline nanocomposites Journal of Material Science: Materials in Electronics 2017 28 7368 7375 10.1007/s10854-017-6425-5

[37] Sairam M Patil MB Veerapur RS Patil SA Aminabhavi TM Novel dense poly(vinyl alcohol)-TiO_2_ mixed matrix membranes for pervaporation separation of water-isopropanol mixtures at 30 °C Journal of Membrane Science 2006 281 95 102 10.1016/j.memsci.2006.03.022

[38] Aminabhavi TM Patil MB Nanocomposite membranes of poly (vinyl alcohol) loaded with polyaniline-coated TiO_2_ and TiO_2_ nanoparticles for the pervaporation dehydration of aqueous mixtures of 1, 4-dioxane and tetrahydrofuran Designed Monomers and Polymers 2010 13 6 497 508 10.1163/138577210X530558

[39] Ayad MM Amer WA Kotp MG Minisy IM Rehab AF Synthesis of silver-anchored polyaniline-chitosan magnetic nanocomposite: a smart system for catalysis RSC Advances 2017 7 30 18553 18560 10.1039/C7RA02575K

[40] Melo-Silveira RF Fidelis GP Costa MSSP Telles CBS Dantas-Santos N In vitro antioxidant, anticoagulant and antimicrobial activity and in inhibition of cancer cell proliferation by xylan extracted from corn cobs International Journal of Molecular Sciences 2011 13 1 409 426 10.3390/ijms13010409 22312261 PMC3269695

[41] Wolkers WF Oliver AE Tablin F Crowe JH A Fourier-transform infrared spectroscopy study of sugar glasses Carbohydrate Research 2004 339 6 1077 1085 10.1016/j.carres.2004.01.016 15063194

[42] Silva FRF Dore C Marques CT Nascimento MS Benevides NMB Anticoagulant activity, paw edema and pleurisy induced carrageenan: Action of major types of commercial carrageenans Carbohydrate Polymers 2010 79 1 26 33 10.1016/j.carbpol.2009.07.010

[43] Song C Yu H Zhang M Yang Y Zhang G Physicochemical properties and antioxidant activity of chitosan from the blowfly Chrysomya megacephala larvae International Journal of Biological Macromolecules 2013 60 347 354 10.1016/j.ijbiomac.2013.05.039 23792633

[44] Tiwari A Sen V Dhakate SR Mishra AP Singh V Synthesis, characterization, and hoping transport properties of HCl doped conducting biopolymer-co-polyaniline zwitterion hybrids Polymers for Advanced Technologies 2008 19 7 909 914 10.1002/pat.1058

[45] Quillard S Louarn G Lefrant S MacDiarmid AG Vibrational analysis of polyaniline: A comparative study of leucoemeraldine, emeraldine, and pernigraniline bases Physical Review B 1994 50 17 12496 10.1103/PhysRevB.50.12496 9975409

[46] Wang PC Dan Y Liu LH Effect of thermal treatment on conductometric response of hydrogen gas sensors integrated with HCl-doped polyaniline nanofibers Materials Chemistry and Physics 2014 144 1–2 155 161 10.1016/j.matchemphys.2013.12.035

[47] Parthibavarman M Vallalperuman K Sathishkumar S Durairaj M Thavamani K A novel microwave synthesis of nanocrystalline SnO 2 and its structural optical and dielectric properties Journal of Materials Science: Materials Electronics 2014 25 730 735 10.1007/s10854-013-1637-9

[48] Rahy A Yang DJ Synthesis of highly conductive polyaniline nanofibers Materials Letters 2008 62 28 4311 4314 10.1016/j.matlet.2008.06.057

[49] Wang N Li J Lv W Feng J Yan W Synthesis of polyaniline/TiO 2 composite with excellent adsorption performance on acid red G RSC Advances 2015 5 27 21132 21141 10.1039/C4RA16910G

[50] Sambaza SS Maity A Pillay K Polyaniline-coated TiO_2_ nanorods for photocatalytic degradation of bisphenol A in water ACS omega 2020 5 46 29642 29656 10.1021/acsomega.0c00628 33251400 PMC7689664

[51] Cui Y Zhang L Lv K Zhou G Wang ZS Low temperature preparation of TiO_2_ nanoparticle chains without hydrothermal treatment for highly efficient dye-sensitized solar cells Journal of Materials Chemistry A 2015 3 8 4477 4483 10.1039/C4TA06679K

[52] Shalan AE Rashad MM Yu Y Lira Cantú M Abdel Mottaleb MSA Controlling the microstructure and properties of titania nanopowders for high efficiency dye sensitized solar cells Electrochemics Acta 2013 89 469 478 10.1016/j.electacta.2012.11.091

[53] Hochbaum AI Yang P Semiconductor nanowires for energy conversion Chemical Reviews Journal 2010 110 1 527 546 10.1021/cr900075v 19817361

[54] Shklovskii BI Efros AL Electronic properties of doped semiconductors 2013 Springer Science & Business Media

[55] Sarkar A Ghosh P Meikap AK Chattopadhyay SK Chatterjee SK Electrical-transport properties of iodine-doped conducting polyaniline Journal of Applied Polymer Science 2008 108 4 2312 2320 10.1002/app.27615

[56] Vellakkat M Kamath A Raghu S Chapi S Hundekal D Dielectric Constant and Transport Mechanism of Percolated Polyaniline Nanoclay Composites Industrial and Engineering Chemistry Research Journal 2014 53 43 16873 16882 10.1021/ie502922b

[57] Badi N Mekala R Khasim S Roy AS Ignatiev A Enhanced dielectric performance in PVDF/Al-Al 2 O 3 core-shell nanocomposites Journal of Materials Science: Materials Eletronics 2018 29 10593 10599 10.1007/s10854-018-9123-z

[58] Bandgar DK Navale ST Vanalkar SA Kim JH Harale NS Synthesis, structural, morphological, compositional and electrical transport properties of polyaniline/α-Fe_2_O_3_ hybrid nanocomposites Synthetic Metals 2014 195 350 358 10.1016/j.synthmet.2014.07.005

[59] Najim TS Salim AJ Polyaniline nanofibers and nanocomposites: Preparation, characterization, and application for Cr (VI) and phosphate ions removal from aqueous solution Arabian Journal of Chemistry 2017 10 S3459 S3467 10.1016/j.arabjc.2014.02.008

[60] Komaba K Goto H Synthesis of polyaniline and polyaniline/fiber composites in geothermal water Journal of Water Chemistry and Technology 2023 45 1 52 62 10.3103/S1063455X23010046

[61] Freitas TV Sousa EA Fuzari GC Jr Arlindo EPS Different morphologies of polyaniline nanostructures synthesized by interfacial polymerization Materials Letters 2018 224 42 45 10.1016/j.matlet.2018.04.062

[62] Liu W Chang YC Zhang J Liu H Wet-spun side-by-side electrically conductive composite fibers ACS Applied Electronics Materials Journal 2022 4 4 1979 1988 10.1021/acsaelm.2c00150

[63] Bhadra J Al Thani NJ Madi NK Al-Maadeed MA Effects of aniline concentrations on the electrical and mechanical properties of polyaniline polyvinyl alcohol blends Arabian Journal of Chemistry 2017 10 5 664 672 10.1016/j.arabjc.2015.04.017

[64] Wang N Cheng K Wu H Wang C Wang Q Effect of nano-sized mesoporous silica MCM-41 and MMT on corrosion properties of epoxy coating Progress in Organic Coatings 2012 75 4 386 391 10.1016/j.porgcoat.2012.07.009

[65] Ćurković L Ćurković HO Salopek S Renjo MM Šegota S Enhancement of corrosion protection of AISI 304 stainless steel by nanostructured sol-gel TiO_2_ films Corrosion Science 2013 77 176 184 10.1016/j.corsci.2013.07.045

[66] Fang B Yan J Chang D Piao J Ma KM Scalable production of ultrafine polyaniline fibres for tactile organic electrochemical transistors Nature Communications 2022 13 1 2101 10.1038/s41467-022-29773-9 PMC9018749 35440125

[67] Santim RH Sanches AO da Silva MJ McMahan CM Malmonge JA Electrically conductive nanocomposites produced by in situ polymerization of pyrrole in pre-vulcanized natural rubber latex Polymer Composites 2022 43 5 2972 2979 10.1002/pc.26591

[68] Moussa MA Rehim MHA Ghoneim AM Khairy SA Soliman MA Dielectric investigations and charge transport in PS-PAni composites with ionic and nonionic surfactants Journal of Physics and Chemistry of Solids 2019 133 163 170 10.1016/j.jpcs.2019.05.026

[69] Sharifian M Kern W Riess G A Bird’s-Eye View on Polymer-Based Hydrogen Carriers for Mobile Applications Polymers (Basel) 2022 14 21 4512 10.3390/polym14214512 36365506 PMC9654451

[70] Galal A Zaki MM Atta NF Samaha SH Nasr HE Electroremoval of copper ions from aqueous solutions using chemically synthesized polypyrrole on polyester fabrics Journal of Water Process Engineering 2021 43 102287 10.1016/j.jwpe.2021.102287

[71] Sule R Mishra AK Nkambule TT Recent advancement in consolidation of MOFs as absorbents for hydrogen storage International Journal of Energy Research 2021 45 9 12481 12499 10.1002/er.6608

[72] Mehranfar A Izadyar M Esmaeili AA Hydrogen storage by N-ethylcarbazol as a new liquid organic hydrogen carrier: A DFT study on the mechanism International Journal of Hydrogen Energy 2015 40 17 5797 5806 10.1016/j.ijhydene.2015.03.011

[73] Mohammadi GA Shaterian M Bahrami H Rasuli R Yavari S Electrospun synthesis of polyaniline and titanium dioxide nanofibers as potential electrode materials in electrochemical hydrogen storage Renewable Energy 2024 226 120439 10.1016/j.renene.2024.120439

